# Whole-genome *in-silico *subtractive hybridization (WISH) - using massive sequencing for the identification of unique and repetitive sex-specific sequences: the example of *Schistosoma mansoni*

**DOI:** 10.1186/1471-2164-11-387

**Published:** 2010-06-21

**Authors:** Julien Portela, Christoph Grunau, Céline Cosseau, Sophie Beltran, Christelle Dantec, Hugues Parrinello, Jérôme Boissier

**Affiliations:** 1 UMR 5244 CNRS-EPHE-UPVD. Parasitologie Fonctionnelle et Evolutive, CBETM. Université de Perpignan, Perpignan, France; 2 Plateforme MGX, Institut de Génomique Fonctionnelle 141, rue de la Cardonille, Montpellier, France

## Abstract

**Background:**

Emerging methods of massive sequencing that allow for rapid re-sequencing of entire genomes at comparably low cost are changing the way biological questions are addressed in many domains. Here we propose a novel method to compare two genomes (genome-to-genome comparison). We used this method to identify sex-specific sequences of the human blood fluke *Schistosoma mansoni*.

**Results:**

Genomic DNA was extracted from male and female (heterogametic) *S. mansoni *adults and sequenced with a Genome Analyzer (Illumina). Sequences are available at the NCBI sequence read archive http://www.ncbi.nlm.nih.gov/Traces/sra/ under study accession number SRA012151.6. Sequencing reads were aligned to the genome, and a pseudogenome composed of known repeats. Straightforward comparative bioinformatics analysis was performed to compare male and female schistosome genomes and identify female-specific sequences. We found that the *S. mansoni *female W chromosome contains only few specific unique sequences (950 Kb i.e. about 0.2% of the genome). The majority of W-specific sequences are repeats (10.5 Mb i.e. about 2.5% of the genome). Arbitrarily selected W-specific sequences were confirmed by PCR. Primers designed for unique and repetitive sequences allowed to reliably identify the sex of both larval and adult stages of the parasite.

**Conclusion:**

Our genome-to-genome comparison method that we call "whole-genome *in-silico *subtractive hybridization" (WISH) allows for rapid identification of sequences that are specific for a certain genotype (e.g. the heterogametic sex). It can in principle be used for the detection of any sequence differences between isolates (*e.g*. strains, pathovars) or even closely related species.

## Background

Massive sequencing, or next-generation sequencing (NGS), has remarkably reduced the cost, time and amount of biological material required for (re-)sequencing of entire genomes. Recently, for instance, whole-genome wide sequence variation in *Caenorhabditis elegans *was assessed comparing Solexa Sequence Analyser reads to a reference genome (strain-to-reference comparison) [1]. In principle, it is possible with this method to identify differences between genomes without a priori knowledge of their location in the genome. This is a fundamental question in many ecological or medical important species. Here, we describe how to identify differences in the DNA sequence of two genomes obtained by a massive parallel sequencing approach (genome-to-genome comparison). We used the method to identify sex specific sequences in the human blood fluke *Schistosoma mansoni*. *S. mansoni *(Trematoda: Digenea) is a gonochoric endoparasite causing a serious human disease called schistosomiasis. Schistosomiasis ranks second only to malaria in terms of parasite induced human morbidity and mortality, with over 200 million people infected worldwide. In schistosomes, sex is determined by sex chromosomes, with female being the heterogametic sex (ZW) and male the homogametic sex (ZZ) [2]. If male and female adult worms show evident phenotypic dimorphism, the larval stages are morphologically indistinguishable making sex-specific infection, crosses and linkage studies extremely difficult. Traditional methods of identification of W-specific sequences have failed to deliver faithful markers [3]. We reasoned that male (ZZ) *vs*. female (ZW) whole genome comparison would enable to identify female specific sequences that are only present on the W chromosome. We split the bioinformatics analysis into two axes: one for the unique sequences and one for the repetitive sequences which allowed us to identify several new classes of female specific repeats and 105 contigs containing unique sequences.

## Results

### Biological material

**Figure 1:**
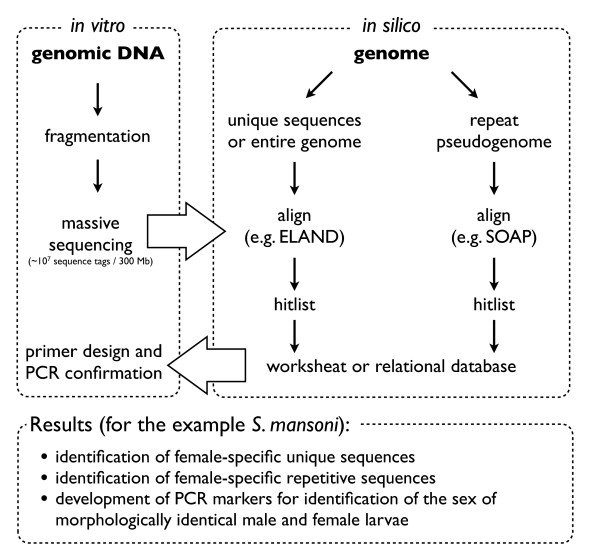
**Schematic representation of the experimental strategy and results for our model**.

**Figure 2:**
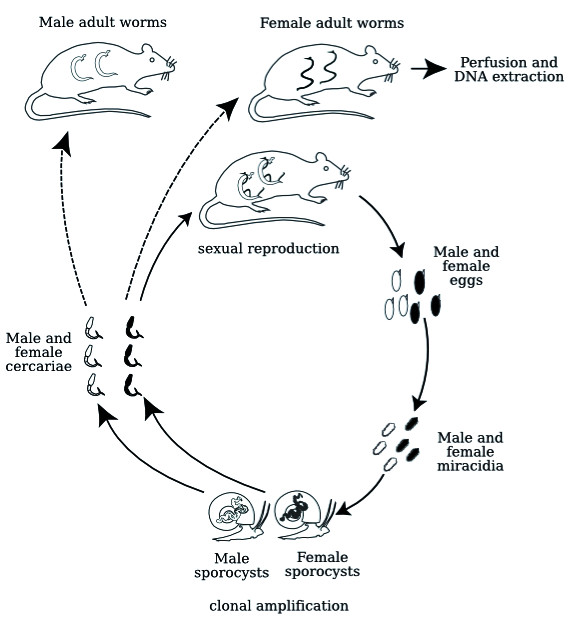
***Schistosoma mansoni *life cycle representing the separated sexes of the parasite**. Sexual reproduction occurs between male and female adult worms in the vertebrate definitive host. Sex determination is syngamic, thus one egg produces either a male or a female larva (called miracidium). This larva actively infects a mollusc intermediate host, transforms into intramolluscan larval stages (called sporocysts) and produces, by clonal multiplication, many unisexual larvae (called cercariae) that will actively infect the vertebrate definitive host and transform into adult worms. For our experimental approach, molluscs were infected with a single miracidium, thus produced either male or female cercariae.

The experimental strategy is outlined in figure 1. In this study we used a *S. mansoni *strain isolated from naturally infected molluscs from Guadeloupe (French West Indies), a Guadeloupean strain of *Biomphalaria glabrata *as intermediate hosts, and the Swiss OF1 mouse strain as final hosts (for the parasite life cycle see figure 2). Methods for mollusc, mouse infections and parasite recovery have been previously described [4]. Briefly, mollusc infection consists in a simple contact in spring water between parasite larvae (miracidia) and molluscs, mouse infection is performed under general anaesthesia and parasite larvae (cercariae) penetrate naturally through the host skin. Finally, parasite recovery is performed by hepatic perfusion of the mouse. Less than 10 μg DNA, in our case from 23 male (5 μg DNA) and 91 female (1.2 μg DNA) adult flukes recovered from mice infected with a single sex and of the same clonal population was extracted using a method adapted to ChIP-Seq but without the immunoprecipitation step [5].

### Sequencing

Solexa sequencing was performed on a Genome Analyzer II (Illumina) by single end sequencing according to the manufacturers protocol. Twenty ng of MNase fragmented DNA from each sample was repaired to generate phosphorylated blunt ends. An adenosine was added to the 3' end of the blunt phosphorylated DNA fragments. Illumina's adapters were ligated to the DNA fragments. Size selection was performed using a 2% agarose gel and a slice was excised at 200 bp corresponding to an insert size of 140 bp. The DNA extracted from the gel was then used as a matrix for 18 cycles PCR using Illumina's PCR primers. Each library was purified and quantified using a DNA1000 Chip on a 2100 BioAnalyzer (Agilent Technologies). These libraries were denaturated using NaOH and then diluted to a final concentration of 2 pM. One hundred μl of these diluted libraries were used for clustering on the Cluster Station using Clustering Kit V.2 (Illumina) and sequencing on the Genome Analyzer using a 36 Cycle SBS Kit V.3 (Illumina). A total of 8,600,198 (309,607,128 bp) and 9,355,380 (336,793,680 bp) sequence reads were produced with GAPipeline 1.3 for the male and female, respectively. This translates to roughly one-fold coverage of the genome, which is sufficient for the described approach.

### Analysis of unique sequences

ELAND was used to align the reads to the reference genome of *S. mansoni *(Puerto Rico) scaffolds, draft version 3.1 (version: 05/08/2008) [6]ftp://ftp.sanger.ac.uk/pub/pathogens/Schistosoma/mansoni/genome/gene_predictions/GFF/S.mansoni_080508.fasta.gz. The algorithm that is used by the ELAND software aligns only to unique sequences in the genome. Other short-read alignment programs can also be used. ELAND has been developed by Anthony J. Cox (Solexa) to align short read sequence to a reference genome. The first 32 bp of each sequence stretch are used to identify each sequence either as perfect match, 1-mismatch or 2-mismatches. Sequences with mismatches above 2 on the first 32 bp are ignored. The coordinates on the genome of repeat sequences (multiple places in the genome) are not given by the software. A total of 65.2% of the female reads and 70.3% of the male reads were located on the genome by the software. As for many species, the *S. mansoni *genome is sequenced but only partially assembled resulting in a high number (19022) of individual scaffolds. Perl scripts were used to split the alignment results into individual files for each scaffold (SeparateElandReads.pl) and aligned reads ("hits") were counted (AnalyzeElandFiles.pl). Perl scripts can be downloaded from http://methdb.univ-perp.fr/cgrunau/methods/Eland2GBrowse.html. This allowed for identification of scaffolds with a low number of hits in the male and high hit counts in the female, *i.e*. female-specific sequences. For visualization in a genome browser, the ELAND output (s_x_sorted.txt) was converted into the classical ELAND format (s_x_eland_result.txt) with a perl script (available on request), and used for generation of wiggle and gff files with FindPeaks 3.1 [7] and CASHX2.0 [8]. Annotation files were uploaded to an in-house Gbrowse server, and female-specific sequences were confirmed by visual inspection of candidate scaffolds. Regions on these scaffolds that showed hits only from the female genome were used for primer design in order to confirm the bioinformatics analysis (see below). The rational behind this approach is that relatively large (size of a scaffold) differences exist between the female and male genome. Since it could be possible that there are only small differences between the two genomes, we repeated the analysis by using the CASHX data in a sliding window of 500 bp with a step size of 250 bp and compared the results with a relational database. Further bioinformatics analysis could include repeat finding and gene annotation. In our case, Tandem Repeats finder [9] was used to investigate the presence of tandem repeats in the female specific contigs. ESTs were obtained from public databases (SchistoDB [10], GeneDB http://www.genedb.org/, GenBank http://www.ncbi.nlm.nih.gov/Genbank/, gene prediction algorithms http://compbio.ornl.gov/tools/pipeline/, http://opal.biology.gatech.edu/GeneMark/eukhmm.cgi were used to test for the presence of putative genes, and MotifScan http://hits.isb-sib.ch/cgi-bin/PFSCAN was employed for prediction of function.

### Analysis of repetitive sequences

As for many eukaryotes, roughly 40% of the *S. mansoni *genome is composed of repetitive sequences. The conventional alignment algorithms cannot use these sequences, and they are in general not considered for analysis. To make use of these repeats, we exploited the repeatmasker database of *S. mansoni *ftp://ftp.tigr.org/pub/data/Eukaryotic_Projects/s_mansoni/preliminary_annotation/homology_evidence/sma1.repeats.gz, added repeats that were available in the literature [11,12], and a tandem repeat (TR266) that was identified with Tandem Repeat finder (see above). This produced a sequence file that is composed of repeats with each repeat occurring only once (repeat pseudogenome). For other genomes, *de-novo *prediction of repeats would be necessary and the NGS data that do not align to the unique sequences could also be assembled to obtain a repeat pseudogenome. The pseudogenome was indexed with 2bwt-builder of soap2.17 [13], and the Solexa fastq files were used for alignment with SOAP. 16.26% (female) and 15.06% (male) of the reads mapped to the pseudogenome, *i.e*. were identified as repeats. Taking into account the above-mentioned unique sequences, this leaves 18.54% (female) and 14.64% (male) unidentified. From the soap output, repeats with at least five hits and for which at least 99% of the total hits occurred in the female genome were used for further analysis (Table 1). Soap files were converted into gff format with a tool of the pass utilities [14] and distribution of hits was visualized with Excel (Microsoft Corp.). In the male genome, hits occurred exclusively on the flanks of the repeats corresponding probably to integration and/or excision sites (Additional file 1, figure S1). Sequences are available at the NCBI sequence read archive http://www.ncbi.nlm.nih.gov/Traces/sra/ under study accession number SRA012151.6.

**Table 1:** Name and number of female and male hits and length of the selected repeats.

repeat	% of total hits on female	Length (bp)	**GenBank acc.nr**.
W1	100.00	482	[J04665.1]
R = 407.2	100.00	711	[GU562605]
W2	100.00	715	[U10109.1]
TR266	99.97	267	[GU562608]
R = 879	99.91	654	[GU562606]
Sm_alphafem1	99.86	338	[U12442.1]
R = 564	99.27	1129	[GU562607]

Repeats were considered as female (W) specific if at least five hits occurred and if at least 99% of the total hits occurred in the female genome. %female = (number of hits obtained with SOAP for the female genome * 100/number of hits obtained with SOAP for the female genome + number of hits obtained with SOAP for the male genome)

### Confirmation of WISH-identified sex-specific sequences

**Figure 3:**
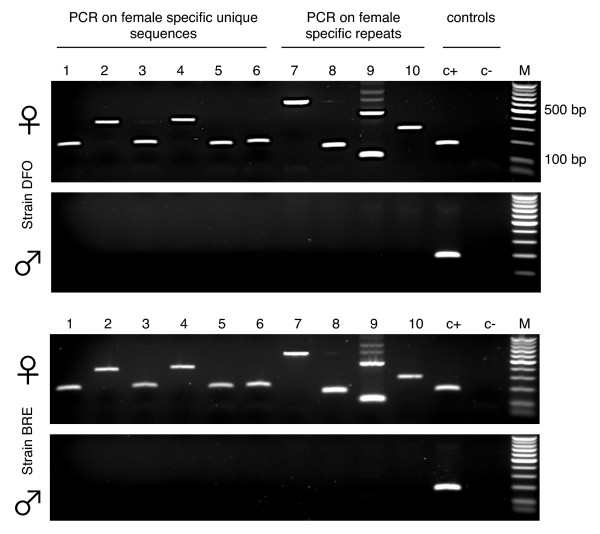
**Ethidiumbromid stained agarose gels (1.5%) with PCR products for typical examples of W-specific primer pairs (SmWSPP1-10)**. Primers and PCR conditions are listed in additional file 2, table S1. Positive control (c+) is a primer pair in the autosomal rhodopsine gene, negative control (c-) is water. M is size marker (100 bp, Promega). Two strains of *S. mansoni *were analysed (DFO (top) and BRE (bottom panel)). On upper part of each panel, PCR products for DNA of female adults, on the bottom part, PCR on DNA of male adults.

PCR were done to confirm the *in-silico *analysis. *Schistosoma mansoni *W specific primers pairs (SmWSPP, Additional file 2 table S1) were designed in the female-specific regions using Primer 3 http://frodo.wi.mit.edu/ and checked for specificity using Primer-Blast http://www.ncbi.nlm.nih.gov/tools/primer-blast/index.cgi. DNA from male and female adult worms was extracted [15] and PCR amplifications were performed in duplicate. PCR reactions were carried out in a total volume of 10 μl containing 1 μl of 10× buffer (450 mM Tris HCl (pH 8.8), 110 mM ammonium sulfate, 45 mM MgCl, 67 mM beta-mercaptoethanol, 44 μM EDTA (pH 8), 1.13 mg/mL BSA) [16], 2 pmol of each oligonucleotide primer, 1 mM of each dNTP (Promega), 0.5 unit of GoTaq polymerase (Promega, Madison, Wisconsin), 1 μl of extracted DNA and DNase-free water. PCR program consisted in an initial denaturation phase at 95°C for 5 min, followed by a suitable number of cycles at 95°C for 30 s, 60°C for 30 s, 72°C for 60 s or less, and a final extension at 72°C for 10 min. Examples of the results are shown in figure 3.

### Ethical note

Our laboratory has received the permit N° A 66040 for experiments on animals from both French Ministry for Agriculture and Fishery (Ministère de l'Agriculture et de la Pêche) and the French Ministry for Higher Education and Research (Ministère de l'Education Nationale de la Recherche et de la Technologie). Housing, breeding and animal care of the mice followed the ethical requirements of our country. The experimenter possesses the official certificate for animal experimentation delivered by both ministries (Décret n° 87-848 du 19 octobre 1987; number of the authorization 007083)

## Discussion

### WISH is a fast and comparably inexpensive alternative for the identification of differences between genomes

The method we describe here is fast: DNA extraction, sequencing and base calling can be done in a week, alignment and sequence analysis depending on the available computing power in another week, and PCR confirmation in a couple of days. The cost of this procedure is comparable to traditional methods such as subtractive hybridization and was in our case less than 3000 Euros. As a result of massive parallel sequencing and straightforward bioinformatics analysis, we identified 180 female-specific contigs and seven repeats.

### WISH identifies unique and repetitive sex-specific sequences

The total genome length of *S. mansoni *is 381,097,121 bp spanning 19,022 contigs [6]. In 1,635 contigs (3,058,411 bp) ELAND found at least one hit in the female genome and no hits in male, and in 1,070 contigs (1,827,612 bp) at least one hit in male but no hits in female. Since about 9,000,000 sequence tags were produced for each genome we expected about 2 hits per 100 bp along the genome, except for the repetitive sequences that are excluded by ELAND. We searched therefore for contigs with at least 1 hit in the female genome and no hit in males. 888 contigs (1,816,279 bp) fulfilled this criteria, but most were small and only 269 were longer than 2 kb (total length 950,812 bp) (Additional file 3, table S2). All 269 contigs were analyzed by visual inspection. The criterion for regarding a contig here as female-specific was the absence or strong under-representation of CASHX hits in the male genome, and presence in the female genome. 105 contigs (436,269 bp) were retained. We then processed the genome in 500 bp windows with a step size of 250 bp searching for fragments with more than 3 hits/500 bp in the female genome and less than one for the male genome. 304 fragments fulfilled this criterion and they were inspected manually. All fragments were dispersed on the genome and no W-specific regions or pseudo-autosomal regions could be identified. Consequently, the W-specific unique regions span at most 950 kb i.e. about 0.2% of the genome. A preliminary sequence analysis did not reveal any sex determination gene.

Seven repeats (R = 407, R = 879, Sm_alphafem1, R = 564, TR266, W1, W2) were identified as female specific. Three of the sequences had been identified before: Sm_alphafem1 [GenBank:U12442.1] was isolated in 1995 by a subtractive hybridization process as female-specific sequence of the alpha retrotransposon family [12]. The copy-number of the Sm_alphafem family of repeats (Smα) was estimated to be 20,000-200,000 [17]. W1 [GenBank:J04665.1] was found by Webster and colleagues and its estimated copy number is 500 [11]. The female-specific repetitive DNA W2 [GenBank:U10109.1] was identified by a PCR-based approach (Representational difference analysis) [12]. One of the newly identified repeats (R = 407) has 95% identity to W2, all other pairwise similarities are around 50%. Characteristics are listed in table 1. Assuming that around 1000 copies exist for each repeat other than Sm_alphafem, then this would correspond to around 10.5 Mb (~2.5% of the genome). Blast against the *S. mansoni *genome (assembly 3.1) allowed for identification of contigs that contain these repeat sequences. All contigs were inspected for presence or absence of male and female NGS hits, and used to complete the list of female-specific contigs resulting in a total of 180 contigs (603,758 bp) (Additional file 3, table S2). WISH is a method to identify sequence differences. These sequences can now be analyzed by other methods.

### WISH-identified W-specific sequences can be used for sex identification

A possible use (and our primary interest) of the genome comparison is the identification of markers that can be amplified by PCR. We designed primers for some contigs and repetitive sequences (Additional file 2, table S1). All tested primer pairs showed the PCR product at the expected size on the adult female but not on the adult male parasite (Figure 3). For SmWSPP 9 (Sm_alphafem1), W-specific PCR products of different size were amplified probably due to the repetitive nature of the target sequence. The same results were obtained on individual parasite larval stages (data not shown). In addition to male-to-female comparison, WISH can be used to identify genetic differences between strains, pathovars or even closely related species opening up a wide range of possible applications. One possible candidate would be *S. japonicum *for which there is a situation similar to our model (draft genome available, female-specific part of the genome unknown). It might be argued that the method requires the genome to be sequenced. This is obviously true, but currently 1001 genomes are completed, for 1279 the draft assembly is available and 1206 genomes are in progress http://www.ncbi.nlm.nih.gov/genomes/static/gpstat.html. These numbers will continue to increase and for most species of medical and ecological importance the genomes will become available. To determine the mode of sex determination is a challenging question for many species [18]. In the case of *S. mansoni*, female-specific markers have been hunted for the last 30 years and 3 female-specific repeat had been identified by classical methods. These repeats were also identified by our approach and served as a positive control. The reason for their earlier discovery is probably that they are the most abundant female-specific sequences in the *S. mansoni *genome (data not shown). The W1 repeat was used for the identification of female larvae, however, experiments in our laboratory and evidence from other labs indicates that the marker could be used for a certain number of generations but sporadically the PCR would amplify also from the male genome [3,19,20]. We do not exclude that the repeat-based PCR markers we present here do not behave similar. Routinely, we use two unique sequences for sex determination, a strategy that works well in our hands. A detailed description is available as additional files and on our webpage http://methdb.univ-perp.fr/cgrunau/methods/sexing_schisto.html

## Conclusions

We show here that using massive sequencing and PCR to detect sex-specific sequences is a reliable and straightforward method to clarify the sex determination issue. The identified markers can be used to identify the sex of individuals in early developmental stages or for adults in species without apparent sexual dimorphism. Sex identification method could also be of clear interest to control the sex in domestic animal reproduction in livestock industry [21,22]. Other applications lie in molecular ecology to identify sex-specific patterns like biased sex-ratio or bias in the dispersal of each sex [23,24]. Naturally, as mentioned above, the method can also be used to detect sequence differences in other scenarios.

## Competing interests

The authors declare that they have no competing interests.

## Authors' contributions

CG, CC, and JB conceived and designed the experiments. CG and CC performed *in silico *analyses. JP and SB experimentally verified *in silico *analyses. HP and CD performed sequencing runs. CG, JB and CC wrote the manuscript. All authors read and approved the final manuscript.

## Supplementary Material

Additional file 1 **Figure S1**. Distribution of SOAP hits along the consensus sequence of female-specific repeats. X-axis: repeat sequence in bp, y-axis: number of hits. Click here for file

Additional file 2 **Table S1**. Characteristics of *Schistosoma mansoni *W chromosome (female) specific primer pairs. Click here for file

Additional file 3 **Table S2**. Female specific contigs (scaffolds of Schistosoma mansoni draft version 3.1.) and available evidence for their identification. All contigs verified by visualization of CASHX hits. Click here for file
