# Supplementary material for: Whole-genome in-silico subtractive hybridization (WISH) - using massive sequencing for the identification of unique and repetitive sex-specific sequences: the example of Schistosoma mansoni
Source: BMC Genomics. 2010 Jun 21;11:387. doi: 10.1186/1471-2164-11-387 (PMC3091631; doi:10.1186/1471-2164-11-387)

W1 female

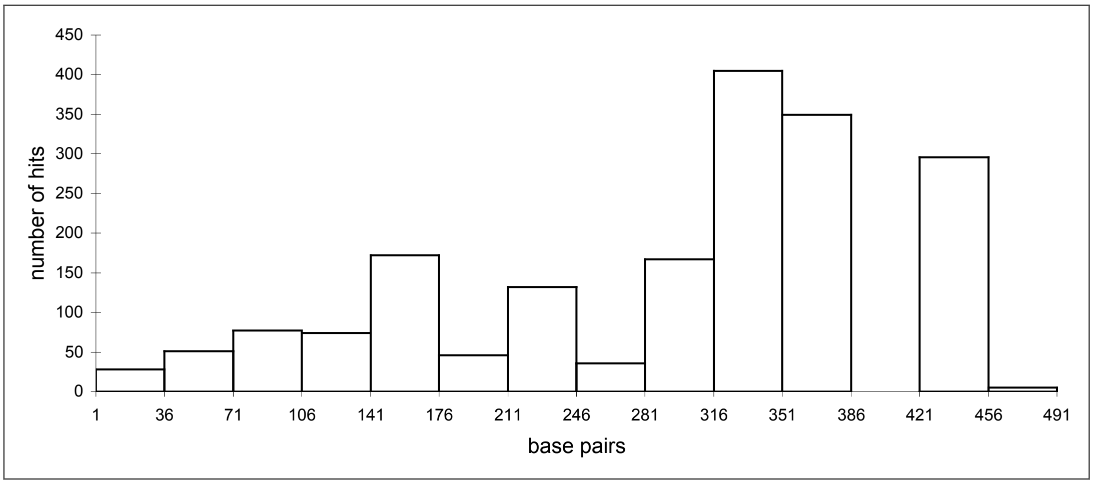

R=407 female

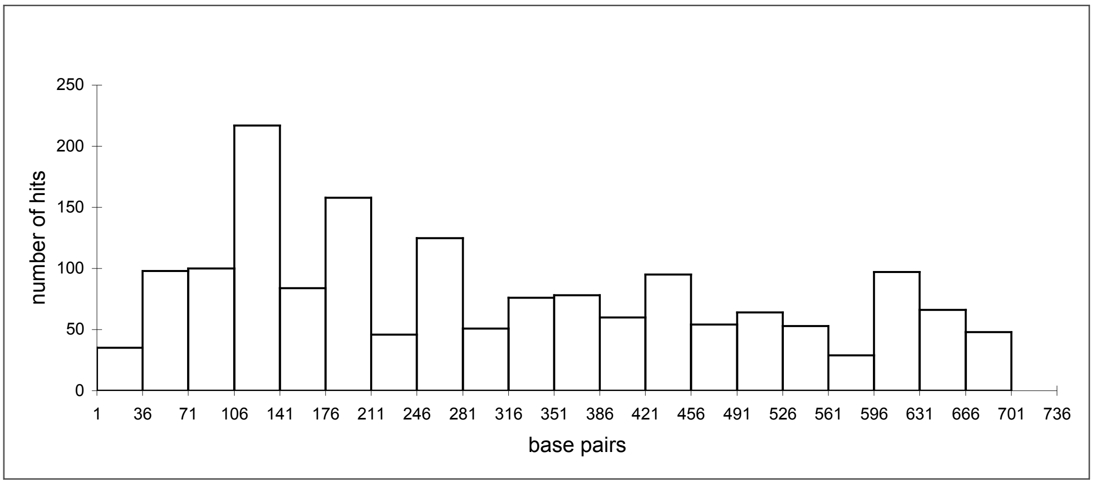

W2 female

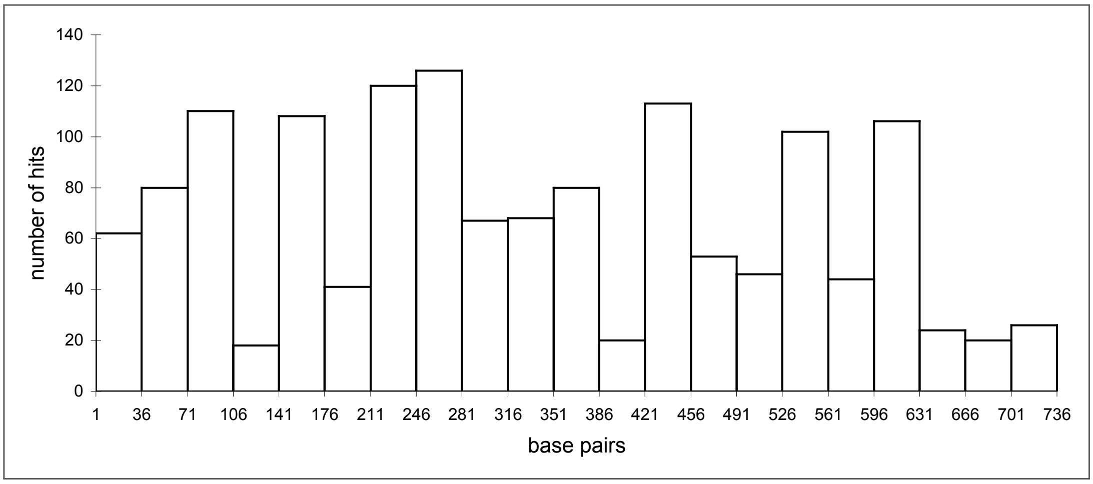

TR266 female

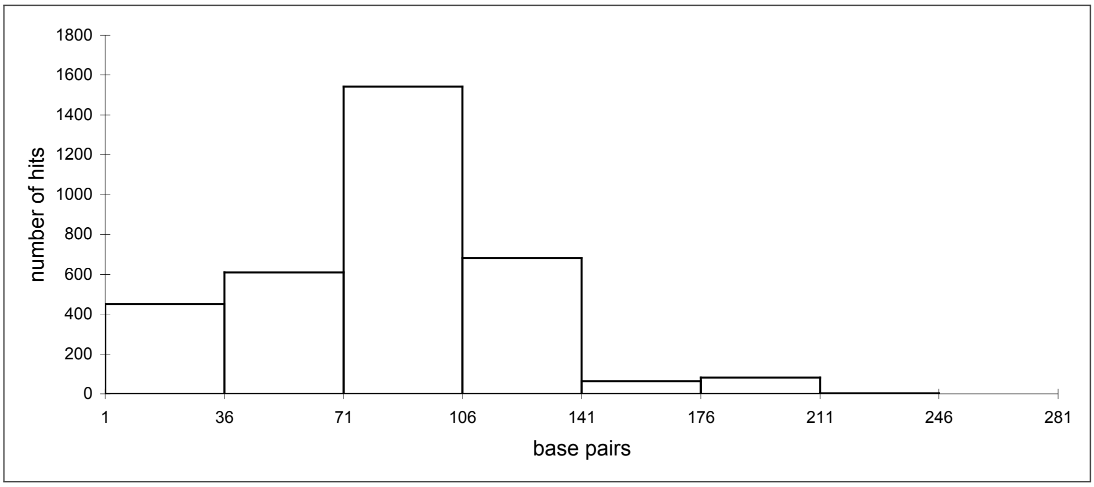

R=879 female

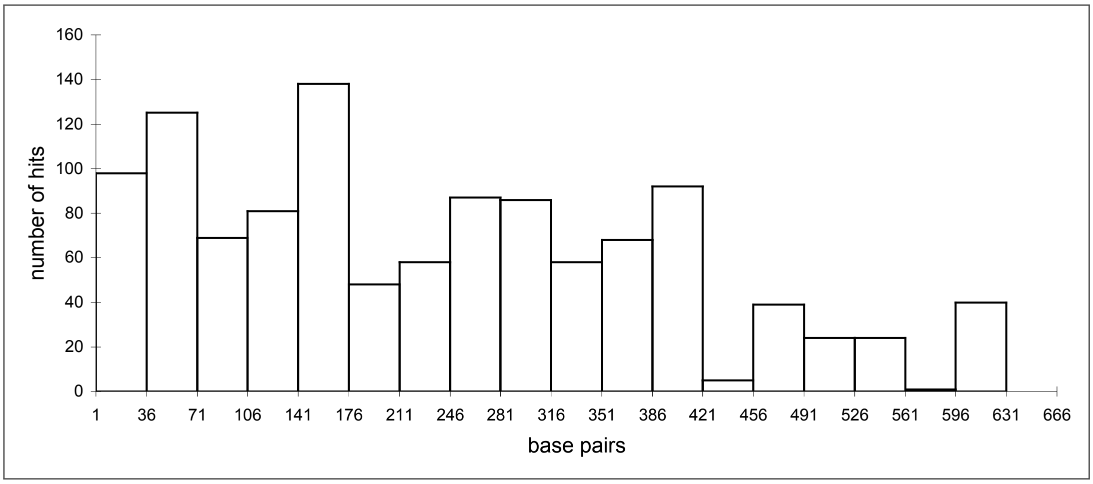

Sm_alphafem1 female

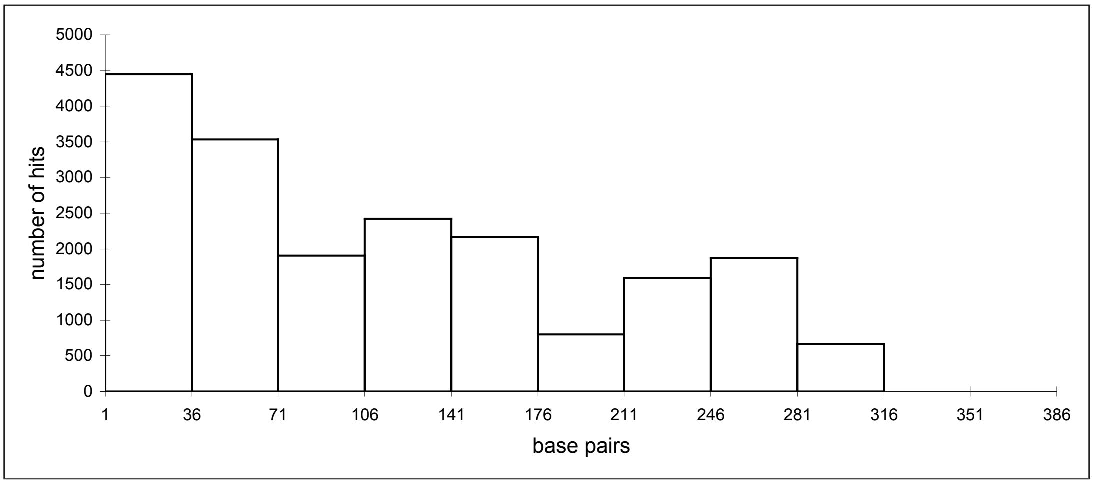

Sm_alpha_female male

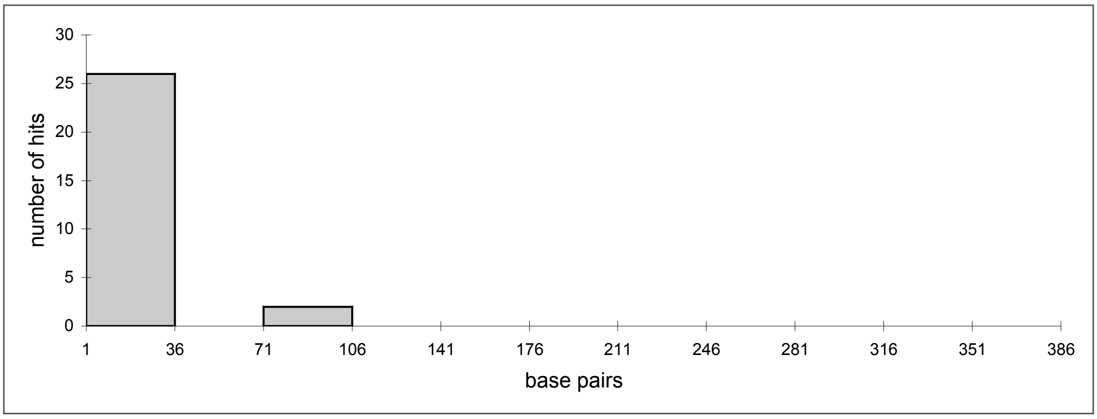

R=564 female

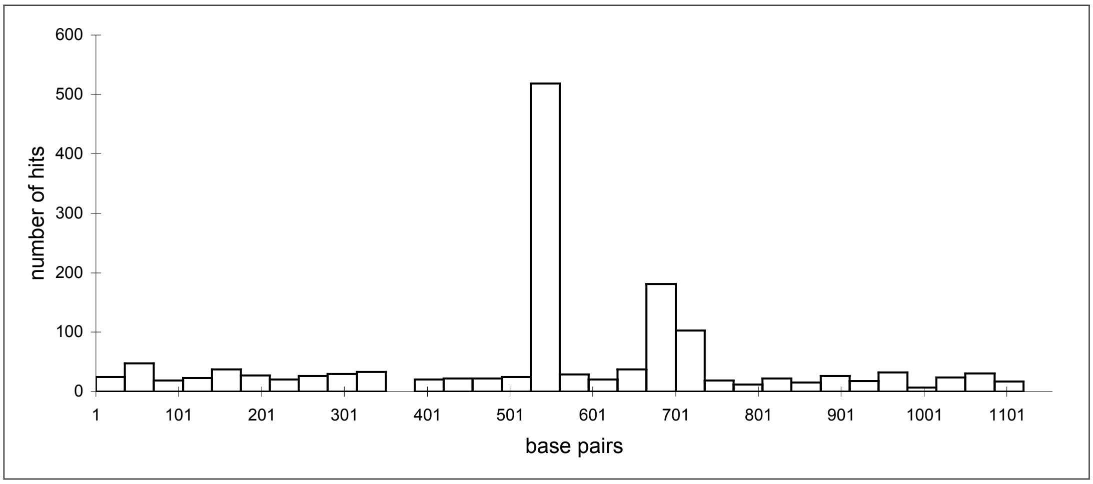

R=564 male

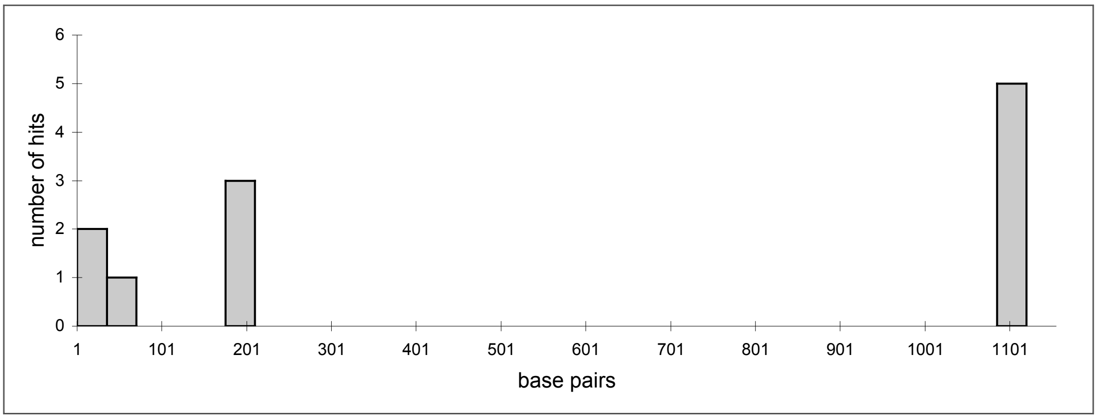

Supplement: Additional file 1 — Figure S1. Distribution of SOAP hits along the consensus sequence of female-specific repeats. X-axis: repeat sequence in bp, y-axis: number of hits. [file 1471-2164-11-387-S1.PDF]
