# Supplementary material for: Whole-genome in-silico subtractive hybridization (WISH) - using massive sequencing for the identification of unique and repetitive sex-specific sequences: the example of Schistosoma mansoni
Source: BMC Genomics. 2010 Jun 21;11:387. doi: 10.1186/1471-2164-11-387 (PMC3091631; doi:10.1186/1471-2164-11-387)
Supplement: Additional file 2 — Table S1. Characteristics of Schistosoma mansoni W chromosome (female) specific primer pairs. [file 1471-2164-11-387-S2.DOC]

| Primer pair | Forward primer | Reverse primer | Product size (bp) | PCR condition* | Sequence information |
| --- | --- | --- | --- | --- | --- |
| SmWSPP 1 | TTTTGGGGATTTATCATCGC | GGCAATAAGGGCTAACCACA | 201 | 1 | Smp_scaff002739 (1708-1908) |
| SmWSPP 2 | CTGTTTCGAATTTCACACTTCA | CATTCACAGTTTGGCGAACA | 365 | 1 | Smp_scaff002739 (2682-3046) |
| SmWSPP 3 | AGGCTGTCCGTAGCAACACT | CGTCGCTTTCTTACTCCTGC | 206 | 1 | Smp_scaff003066 (1027-1232) |
| SmWSPP 4 | TCGTGTGACTCGCTTGTAGC | ACTTCAGGGATGCCAAACTG | 393 | 1 | Smp_scaff004478 (1790-1995) |
| SmWSPP 5 | CAGTTTGGCATCCCTGAAGT | AGCAGTGCACACCGAAAAC | 201 | 1 | Smp_scaff004478 (2163-2563) |
| SmWSPP 6 | GGCAAAAGAAAAACCACCAA | GGTGACGCCTAGGACACAAT | 210 | 1 | Smp_scaff011102 (858-1067) |
| SmWSPP 7 | TCTACTGCTGATTGGTTGCG | TGCACAATCACAGCTCCTTC | 610 | 2 | R=407 ** (261-870, 972-1581, 1683-2292) |
| SmWSPP 8 | GTGACCGTGTTTTGGTGTTG | GTTATGAGGGTTGACCACGC | 176 | 2 | R=879 ** (241-416) |
| SmWSPP 9 | TGCACAAGTGAGTGGCTGTGGG | TGGATGTACCTGCATCCCGTGT | 120 | 2 | Sm_alphafem1; GenBank U12442.1 |
| SmWSPP 10 | CAAGCTCGCGTACGATGATA | GCTTCACGTGAGTGGTGAGA | 295 | 2 | R=564 ** (489-783) |
| Rhodop 3 | GACGGCCACACTAAAG | AGTAAAATGGTCACTGCTAT | 177 | 1 | Smp_scaff001984 (49840-50016) |

Table S1. Characteristics of *Schistosoma mansoni* W chromosome (female) specific primer pairs.

* Condition 1: 25 cycles; Elongation time 60s; Annealing temperature 60°C. Condition 2: 20 cycles; Elongation time 30s; Annealing temperature 60°C

** ftp://ftp.tigr.org/pub/data/Eukaryotic_Projects/s_mansoni/preliminary_annotation/homology_evidence/sma1.repeats.gz (Najib M. El-Sayed, personal communication)
